# Vaginal Repair of Post-hysterectomy Vesicovaginal Fistula With a Martius Flap: A Case Report

**DOI:** 10.7759/cureus.110195

**Published:** 2026-06-03

**Authors:** Daniel O Silva, Rafael Almeida, Diogo Lima, Margarida B Paiva, Ricardo Sarmento

**Affiliations:** 1 Obstetrics and Gynecology, Unidade Local de Saúde do Arco Ribeirinho, Barreiro, PRT; 2 Urology, Unidade Local de Saúde do Arco Ribeirinho, Barreiro, PRT; 3 Gynecology, Unidade Local de Saúde do Arco Ribeirinho, Barreiro, PRT

**Keywords:** chronic vesicovaginal fistula, martius flap, quality of life improvement, surgical and clinical management, urinary incontinence

## Abstract

A vesicovaginal fistula (VVF) is a devastating condition often resulting from gynecological surgery. We present the case of a chronic VVF treated via a vaginal approach with a Martius flap.

A 38-year-old woman presented with three years of urinary incontinence following a total abdominal hysterectomy. Diagnosis of a VVF was confirmed by physical exam, methylene blue test, and cystoscopy. The patient underwent vaginal repair with a Martius flap.

The Martius flap procedure restored urinary continence and improved the patient's quality of life. This case highlights the importance and surgical steps of the Martius flap for the repair of VVF.

## Introduction

A vesicovaginal fistula (VVF) is an abnormal connection between the bladder and the vagina, constituting a major complication in obstetrics and gynecology. In developed countries, VVF most frequently occurs after gynecological surgery, particularly after total abdominal hysterectomy. In contrast, prolonged obstructed labor is the predominant cause in underdeveloped regions. Post-operative fistulas typically manifest approximately 10 days after surgery, whereas radiation-induced fistulas may develop years later [1]. The primary symptoms of VVF include continuous urinary leakage, which may greatly diminish quality of life, as well as recurrent urinary tract infections and vaginal irritation or inflammation. Recognition of these characteristic symptoms supports an accurate clinical diagnosis.

VVFs are classified according to several criteria to facilitate clinical application. Anatomically, they are divided into supratrigonal, trigonal, and infratrigonal types. Size-based classification includes small (≤0.5 cm), medium (0.6-2.4 cm), and large (≥2.5 cm) fistulas [2,3]. Etiologically, VVFs are categorized by their origin, with common causes including gynecological surgery, obstructed labor, chronic disease, radiotherapy, and failed repairs. Simple fistulas are generally small and solitary, whereas complex fistulas, often associated with chronic disease or radiotherapy, tend to be larger or recurrent.

Surgical repair via the vaginal route is the preferred treatment for chronic VVF because it reduces complications, pain, blood loss, and hospital stay. Contraindications include fistulas involving adjacent organs such as the ureters or bowel. Vaginal repair techniques include the Latzko colpocleisis, layered closure, and the Martius flap, with selection guided by fistula characteristics [2,3]. The Latzko colpocleisis is typically indicated for small, proximal, post-surgical, and post-radiation-induced VVFs, particularly recurrent cases, and achieves continence rates exceeding 90% at 12 months. Layered closure is suitable for small, simple fistulas and yields results comparable to those of other methods, with minimal complications. The Martius flap is reserved for larger, complex fistulas and has a high success rate in restoring function. In-depth review of fistula size, location, etiology, and complexity is vital for optimal treatment selection [2-6].

The Latzko colpocleisis is indicated in patients with post-hysterectomy status and sufficient preoperative vaginal vault length. The procedure entails denuding approximately 1 cm of the vaginal wall surrounding the fistula, without excising the bladder fistulous tract, followed by the sagittal closure of the vaginal wall. Although this technique results in minimal blood loss, it may shorten the vaginal canal and lead to sexual dysfunction [2,3].

A layered closure is a simple method for repairing small, simple fistulas. First, the bladder is separated from the vaginal mucosa. After excising the fistulous tract, the bladder is closed with interrupted, delayed absorbable sutures in two layers. The final step is closing the vaginal wall with interrupted, delayed absorbable sutures. The main difference from the Latzko procedure is the mucosal excision of the fistulous tract [2].

The Martius flap treats complex fistulas. The procedure uses a 5-6-cm-long and 2-3-cm-wide fat pad from the labium majus, which is elevated and tunneled subcutaneously into the vagina to serve as a vascular flap between the bladder and vagina. The perineal branch of the internal and external pudendal arteries supplies the flap [5,6].

The surgical approach is selected based on clinical evaluation, fistula complexity, etiology, recurrence status, and the duration of urinary incontinence.

This report presents the case of a woman of African origin with a history of multiple pelvic laparotomic surgeries in her home country, who presented to the gynecology clinic with chronic urinary incontinence. The true incidence of VVF in Portugal is unclear, as social stigma often leads to underreporting. The leading cause is previous pelvic surgery, followed by pelvic radiotherapy.

This case details the successful management of a complex post-hysterectomy VVF using a vaginal approach with a Martius flap, stressing the diagnostic process and surgical technique.

## Case presentation

A 38-year-old woman of African origin presented with a three-year history of involuntary urinary incontinence, which began after a total abdominal hysterectomy performed in her home country for abnormal uterine hemorrhage due to a leiomyomatous uterus. The condition severely impacted her quality of life, preventing her from working and engaging in sexual activity. She had a history of three prior cesarean sections.

Clinical examination revealed a VVF measuring approximately 15 mm in diameter in the anterior vaginal cul-de-sac, located anterior to the vaginal dome. A high volume of urine was observed leaking from the fistula. The diagnosis was confirmed by filling the bladder with methylene blue dye, which was subsequently observed leaking into the vagina.

A ureterocystoscopy showed no urethral lesions and identified a centimetric, infratrigonal fistula on the bladder floor. Significantly, the ureteral meatuses were not involved and had normal urinary output. A pelvic computed tomography (CT) scan confirmed the absence of any other fistulous tracts, ruling out uretero-vaginal or bowel involvement.

The patient underwent a vaginal repair of the VVF with a Martius flap. She was placed in the lithotomy position for the procedure.

**Figure 1:**
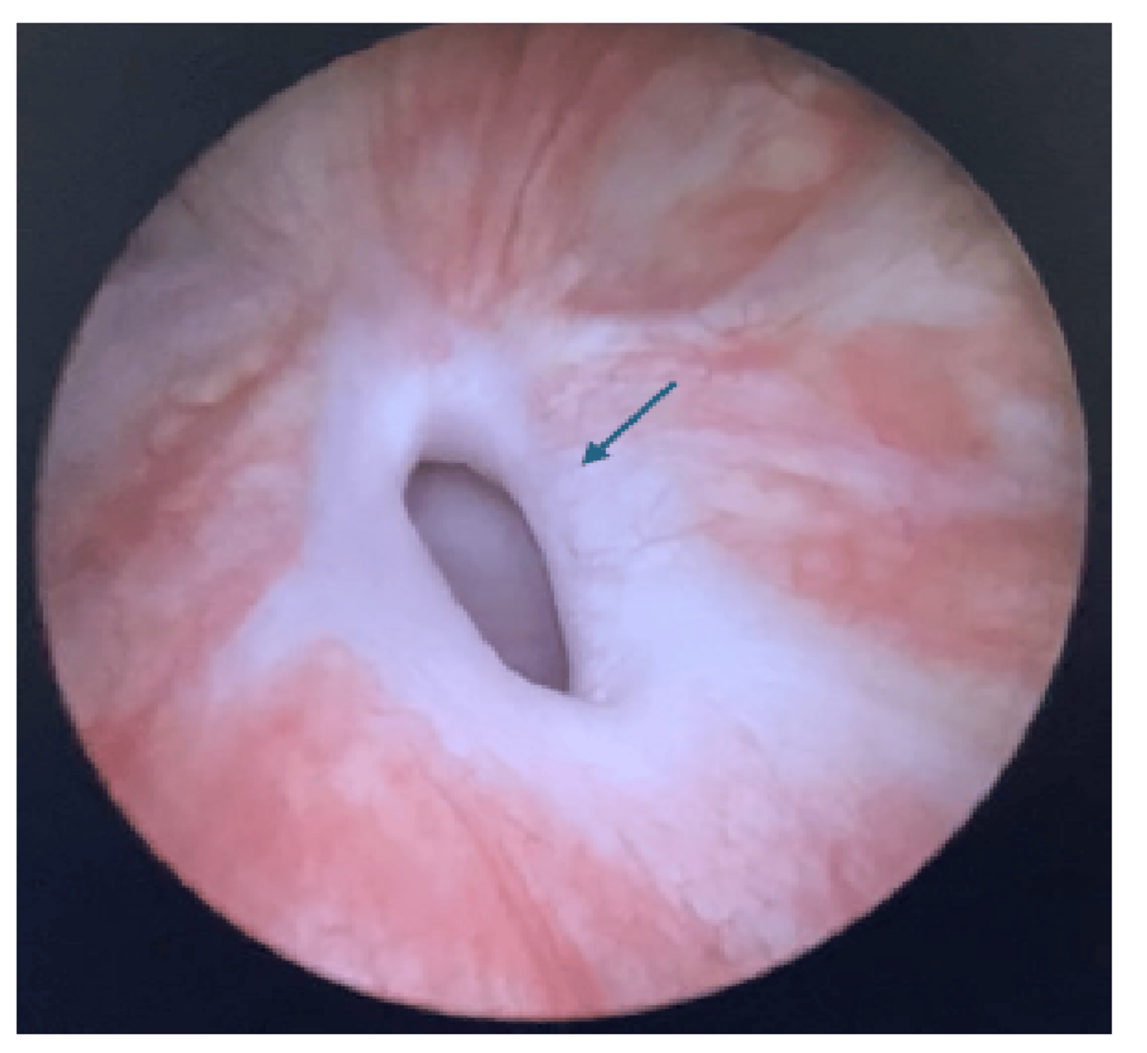
Cystoscopy This image shows the intra-operative cystoscopy view of the VVF. The borders of the fistula tract appear to be surrounded by fibrotic tissue, indicative of its chronic nature (arrow). VVF: vesicovaginal fistula

The procedure began with cystoscopy to confirm the location and size of the fistula and to ensure that the ureters were not involved. Following this, a Foley catheter was inserted (Figure 1).

**Figure 2:**
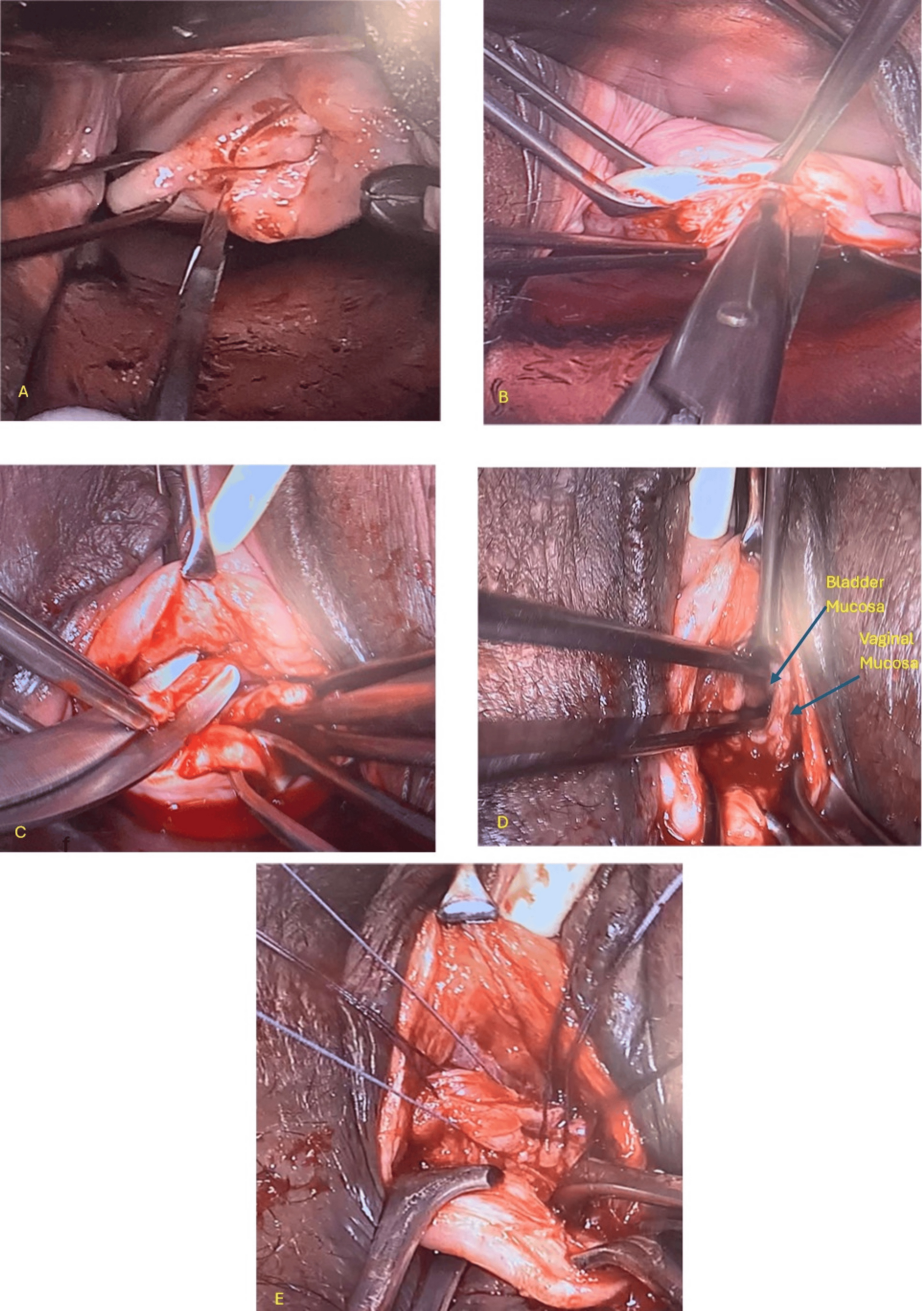
Excision of the fistulous tract This figure illustrates the key steps of the anatomical repair. (A) An ovoid incision is made in the vaginal mucosa around the fistula. (B) Deep excision of the fistulous tract is performed using Metzenbaum scissors until the healthy bladder mucosa is reached. (C) Fibrous tissue is excised, and the margins of the tissue are freshened to prepare for suturing. (D) The healthy bladder mucosa is visible after the debridement. (E) The bladder mucosa is closed with a single layer of interrupted stitches using Polyglactin 3.0 suture.

The next step was the anatomical repair. A circumferential incision was made around the fistula to dissect the bladder and vaginal walls. Fibrotic tissue was carefully removed from the margins of both the anterior and posterior vaginal flaps to ensure a proper seal. The bladder epithelium was then closed in a single layer using interrupted absorbable sutures. The closure's integrity was verified by instilling methylene blue into the bladder, which confirmed a watertight seal (Figure 2).

**Figure 3:**
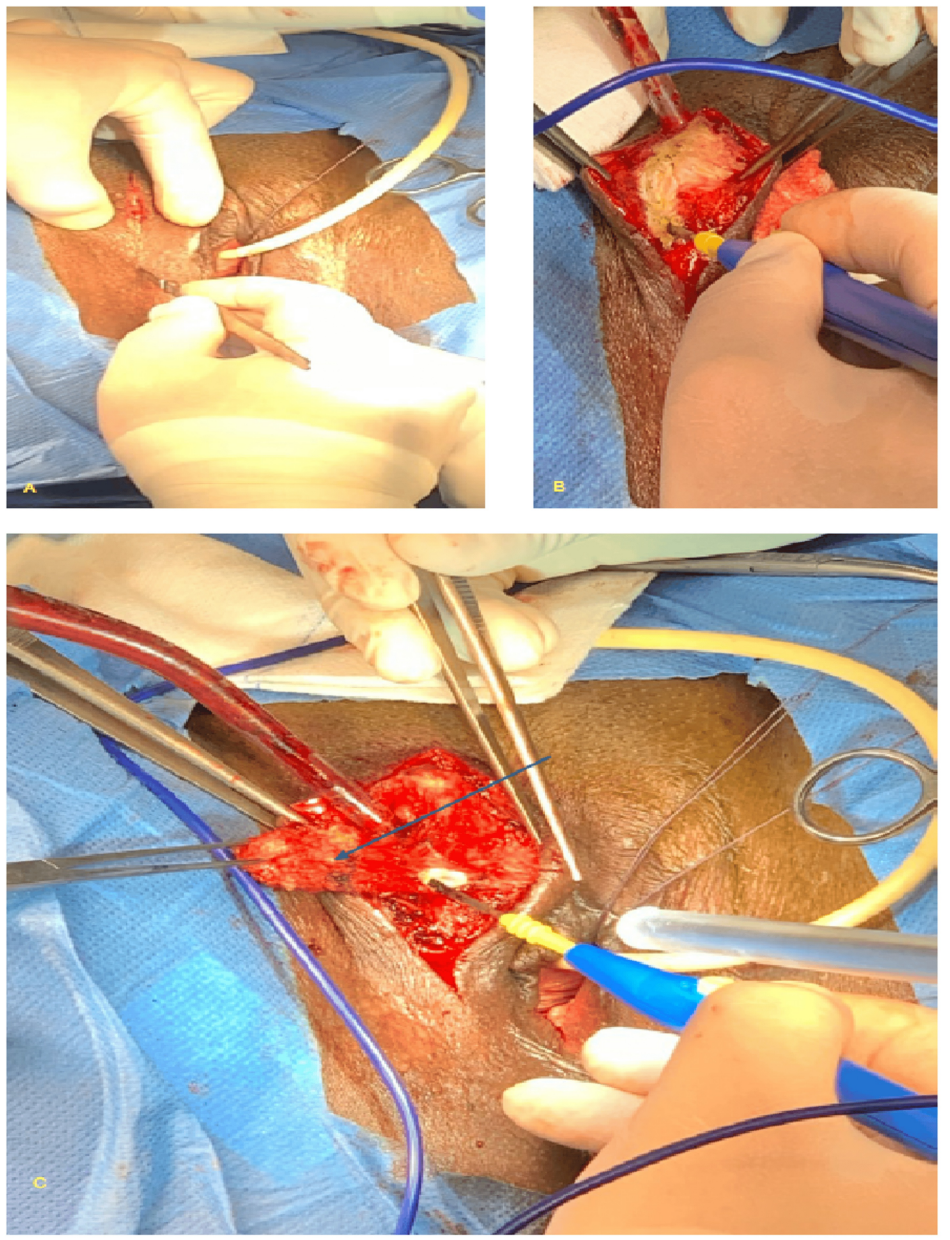
Martius flap This figure details the harvesting of the Martius fat pad. (A) A vulvar skin incision is made lateral to the bulbocavernosus muscle. (B and C) The Martius fat pad is carefully dissected, with the vascular pedicle from the internal pudendal artery clearly visible (arrow), ensuring its blood supply is maintained.

For the Martius flap, an 8-cm lateral incision was made on the right labium majus to expose the fibrofatty pad. This pad was dissected from anteromedial to posterolateral, resulting in a flap approximately 6×3 cm in size. Care was taken to preserve the anteromedial pedicle, which contains the essential blood supply from the internal and external pudendal arteries. The flap was checked to ensure that its length was sufficient to reach the fistula site without tension (Figure 3).

**Figure 4:**
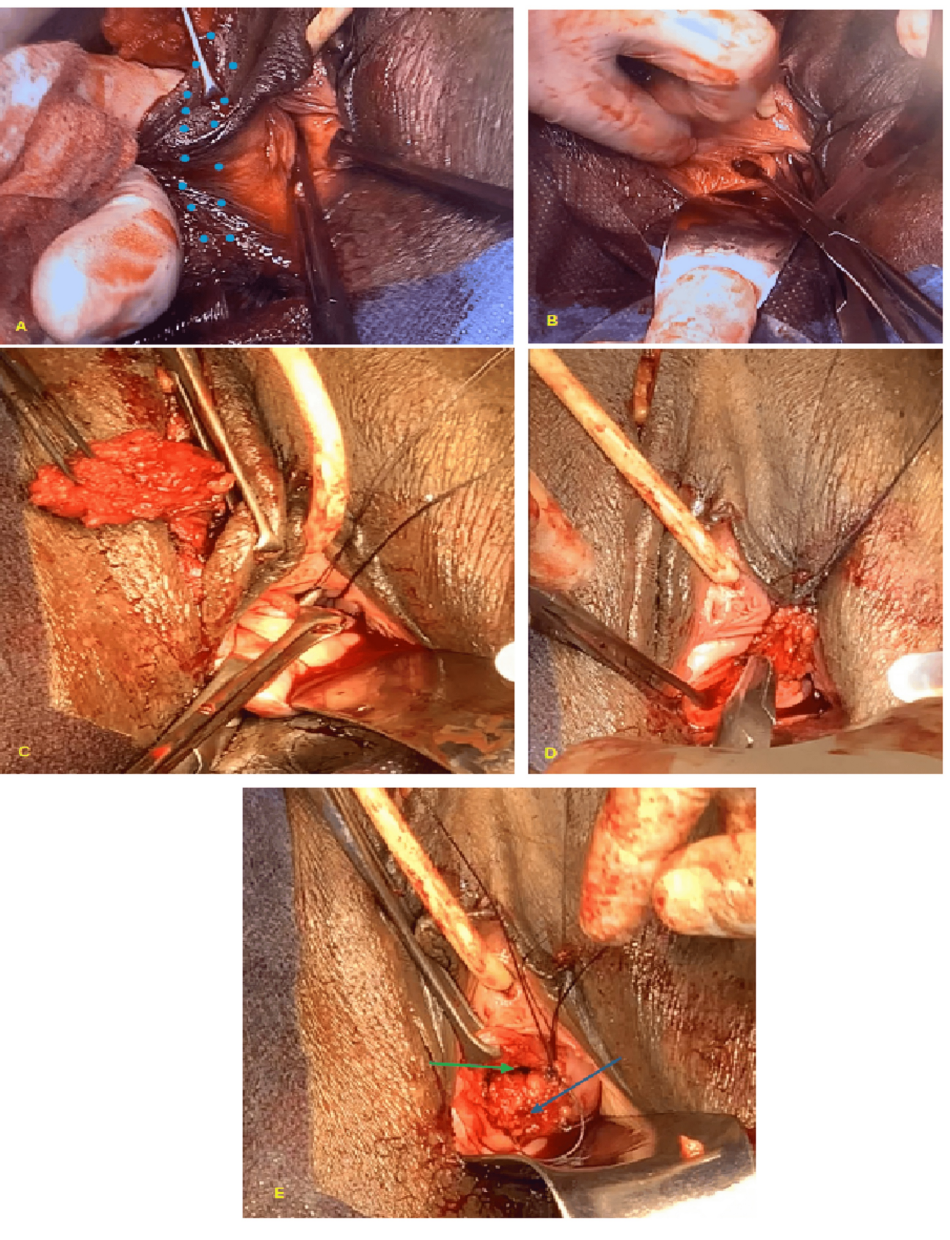
Graft placement This figure demonstrates the final steps of positioning the Martius flap. (A) A tunnel is created by blunt dissection, traveling from the vulvar incision under the bulbospongiosus muscle and beneath the vaginal epithelium. Dotted blue points mark the position of the bulbospongiosus muscle. (B) An opening in the vaginal mucosa is made to allow access to the tunnel. (C) The Martius fat pad is passed through the tunnel, which is made wide enough to prevent vascular compression and potential graft necrosis. (D) The Martius flap is placed and positioned between the repaired fistulous tract and the vaginal mucosa. (E) The graft (Martius fat pad pointed with the blue arrow) is secured to the vaginal muscularis (green arrow) with loosely placed single stitches using Polyglactin 3.0 to prevent tension.

After harvesting the fat pad, it was tunneled under the bulbospongiosus muscle and brought into the vagina. The flap was positioned and fixed between the repaired bladder and the vaginal mucosa using single, tension-free, absorbable sutures. The vaginal mucosa was then closed with interrupted stitches (Figure 4).

Closure of the vulvar skin incision was performed using interrupted subcutaneous sutures and a continuous intradermal suture. A final cystoscopy was carried out to confirm the successful closure of the fistula and to ensure normal urinary output from the ureteral meatuses. A Foley catheter was maintained for three weeks to prevent excessive intravesical pressure and to facilitate optimal healing.

## Discussion

The patient was re-evaluated two months post-surgery and was found to be fully recovered with complete urinary continence. She was able to resume her social life, sexual activity, and work.

The present case highlights the successful application of the Martius flap for the repair of a VVF following hysterectomy. Consistent with previous reports, hysterectomy remains a leading cause of VVF in developed countries [7,8]. Our diagnostic approach, using methylene blue and cystoscopy, aligns with recommendations outlined in previous papers [1,7].

A variety of surgical techniques have been described for the management of VVF. The Martius labial fat pad interposition, as first described in 1928, is particularly indicated for complex or recurrent cases in which local tissue is compromised [9]. Several studies support the efficacy of this technique and demonstrate that patients undergoing Martius flap interposition had high healing rates and lower recurrence. It was also reported that favorable continence and success rates were achieved with the Martius flap in complex fistulas [10].

Alternative techniques, such as the Latzko colpocleisis procedure, are preferred for small, apical, or recurrent fistulas, with reporting continence rates exceeding 90% [11]. However, the Latzko technique may shorten the vaginal canal, which can be a disadvantage for younger women aiming to preserve sexual function.

Recent systematic reviews recommend the Martius flap as a valuable surgical option for complex fistulas, given its ability to provide vascularized tissue and reduce the risk of recurrence [12]. The successful outcome in our case reinforces these findings and underscores the importance of individualized patient assessment and a tailored surgical approach based on the complexity of the fistula and patient preferences.

## Conclusions

This case report confirms efficacy and supports the Martius flap as a first-line option for treating chronic, complex VVF, particularly those resulting from gynecological surgery. Following a thorough diagnostic work-up that included cystoscopy and CT to rule out the involvement of adjacent structures, the Martius flap provided a crucial, highly vascularized tissue barrier, successfully bridging the anatomical defect and addressing the poor tissue quality often associated with complex and recurrent fistulas. The procedure yielded complete restoration of urinary continence and a significant improvement in the patient's quality of life, thereby supporting the Martius flap as a first-line surgical option for large chronic post-hysterectomy VVFs in which tissue interposition is vital for durable repair.
